# Could Renal Vascular Variations Be Associated with Resistant Hypertension? A Single-Center Study

**DOI:** 10.3390/jcm14041268

**Published:** 2025-02-14

**Authors:** Stefan Naydenov, Margarita Jekova, Emil Manov, Nikolay Runev

**Affiliations:** Department of Internal Diseases “Prof. St. Kirkovich”, Medical University of Sofia, 1431 Sofia, Bulgaria; jekovamargarita@yahoo.com (M.J.); doctor_emil_manov@abv.bg (E.M.); nrunev@abv.bg (N.R.)

**Keywords:** renal, vascular, anatomic, variations, resistant, hypertension

## Abstract

**Background**: Anatomical variations in renal vessels are common in humans. However, the clinical significance of these congenital vascular abnormalities remains incompletely understood. This study aimed to assess the prevalence and characteristics of renal vascular variants in patients with arterial hypertension (HTN) and their association with the development of resistant HTN. **Methods**: We screened 3762 consecutive hypertensive patients hospitalized in our clinic and identified 128 (3.4%) with resistant HTN. These patients were matched with 128 hospitalized patients with controlled HTN. All participants underwent contrast-enhanced computed tomography imaging of the kidneys, including renovasography. **Results**: Anatomical renal vascular variations were identified in 64 (25%) of the 256 participants: 49 (38.3%) of the 128 patients with resistant HTN and 15 (11.7%) of the 128 patients with controlled HTN (*p* < 0.001). Among patients with renal vascular abnormalities, 76.6% had resistant HTN, while 23.4% had controlled HTN (*p* < 0.001). A regression analysis demonstrated a strong association between the presence of renal vascular variants and the development of resistant HTN, with an odds ratio of 4.67. **Conclusions**: Anatomical renal vascular variations were found to be common among hypertensive patients in this study and were strongly associated with the development of treatment-resistant HTN.

## 1. Introduction

Anatomical variations in the renal vessels, both arterial and venous, are among the most common vascular abnormalities in humans, with incidence rates ranging from 4% to >60% in the renal arterial system and from 8.0% to ~40% in the venous system, depending on factors such as ethnicity, investigative methods (computed tomography, magnetic resonance imaging, surgical, or pathoanatomical data), and inclusion/exclusion criteria [1,2,3,4,5]. The prevalence of accessory arteries is reported to range between 3% and 30% unilaterally and up to 10% bilaterally in various studies [1,4,6,7,8]. Accessory veins are more commonly found in the right renal vein, with a prevalence of around 16%, compared to the left renal vein (approximately 2%) and other types of vascular malformations are less common [1,5,9,10].

Abnormalities in the development of renal vasculature encompass a highly heterogeneous group of vascular variations, and the clinical significance of many of these abnormalities remains undefined [1,4,11,12,13]. The identification of malformations in the renal vascular system is critical when planning interventions such as renal sympathetic denervation, aneurysm repair, renal transplantation, and other procedures [1,2,6,11,12]. Certain vascular anomalies may also contribute to conditions such as hydronephrosis, varicocele, and orthostatic proteinuria [1,4,9,10,12].

One important unresolved issue is the role of renal vascular variations in the development of arterial hypertension (HTN) [1,4,11,12,13]. Renal vascular diseases are well-established causes of secondary (renal vascular) HTN [14,15,16]. Certain conditions such as atherosclerotic renal artery disease, fibromuscular dysplasia, and specific types of vasculitis, are strongly associated with the development of HTN, which is often refractory to standard pharmacological treatment [14,15]. However, the role of anatomical renal vascular variations in the development of high blood pressure (BP), particularly resistant HTN, remains unconfirmed [4,5,12,13].

Given the relatively high prevalence of these abnormalities in the general population, we conducted this study to assess the overall prevalence and types of renal vascular abnormalities in patients with high BP, as well as their association with the development of unmanageable hypertension.

## 2. Materials and Methods

We performed a retrospective, observational, and non-interventional study. Initially, we screened 3762 hypertensive patients hospitalized at our clinic between 1 July 2018 and 27 March 2024 for resistant HTN. The inclusion criteria were as follows:

1. Individuals aged 18 years or older;
2. A verified diagnosis of “Arterial Hypertension” according to the diagnostic criteria outlined in the 2024 European Society of Cardiology (ESC) and 2023 European Society of Hypertension (ESH) Guidelines [14,15];
3. Office systolic blood pressure (SBP) ≥ 140 mmHg and/or office diastolic blood pressure (DBP) ≥ 90 mmHg persisting despite ≥1 month of therapy with either optimal or maximum-tolerated doses of at least three medications, including a renin–angiotensin–aldosterone system (RAAS) inhibitor, a calcium channel blocker (CCB), and a thiazide/thiazide-like diuretic;
4. The verification of poorly controlled blood pressure through 24 h ambulatory blood pressure monitoring (ABPM);
5. Evidence of patient compliance with treatment regimens, achieving ≥80% adherence during the prescribed period.

The study’s exclusion criteria included the following:

1. Achieved target BP levels with fewer than three antihypertensive drugs;
2. Maintenance of SBP ≥ 140 mmHg and/or DBP ≥ 90 mmHg despite treatment with ≥3 drugs but not at optimal doses or/and not including the combination of RAAS blockers + CCB + thiazide/thiazide-like diuretic, or with a treatment duration of <1 month;
3. Documented cases of secondary hypertension resulting from renal artery involvement (atherosclerotic, fibromuscular, or vasculitic), renal parenchymal disease, or other underlying endocrinological, metabolic, cardiovascular, or related conditions;
4. Presence of clinical conditions and/or comorbidities that rendered the planned instrumental investigations impractical.

After applying the inclusion and exclusion criteria, 128 (3.4%) of the 3762 patients were identified as having drug-resistant hypertension: 63 (49.2%) men and 65 (50.8%) women, *p* = 0.860.

Using propensity score matching, we paired the 128 patients with resistant hypertension with 128 hypertensive patients hospitalized at our clinic during the study period who had achieved target BP levels. The control group was required to meet the following inclusion criteria:

1. Office SBP between 120 and 129 mmHg;
2. Office DBP between 70 and 79 mmHg;
3. Mean 24 h ABPM < 130 mmHg for SBP and <80 mmHg for DBP;
4. Mean daytime ABPM < 135 mmHg for SBP and <85 mmHg for DBP;
5. Mean nighttime ABPM < 120 mmHg for SBP and <70 mmHg for DBP;
6. Blood pressure control was achieved using ≤3 drugs from different classes.

To assess the prevalence and types of anatomical variations in kidney vasculature, all participants underwent contrast-enhanced computed tomography (CT) imaging of the kidneys, including renovasography. Additional instrumental investigations included electrocardiography (ECG), transthoracic echocardiography, and routine laboratory tests.

From hospitalization records and other available medical sources we derived information about demographic characteristics, medical history (e.g., complaints, cardiovascular risk factors, comorbidities, and treatments), and findings from clinical, instrumental, and laboratory assessments. This information was used for cardiovascular risk assessments following the 2021 ESC Guidelines on cardiovascular prevention, along with the 2024 ESC and 2023 ESH Guidelines for hypertension management [14,15,17].

Our study adhered to the ethical standards set by the 1964 Declaration of Helsinki and its subsequent amendments, as well as guidelines for good clinical practice and relevant local regulations. The study was registered on ClinicalTrials.gov on 7 February, 2024, with the reference number KPVB0001RH. Approval from a local ethics committee was not necessary according to the Bulgarian regulatory authorities because our clinical research was observational, retrospective, and non-interventional. All participants provided signed informed consent at the time of hospital admission, agreeing to undergo examinations and treatments in accordance with the proposed diagnostic and treatment plans.

### Statistical Analysis

For data processing and statistical analysis, we utilized IBM SPSS Statistics version 26.0 (SPSS Inc., Chicago, IL, USA). Categorical variables were presented as absolute numbers with corresponding percentages (%), and differences were assessed using the chi-square test. Continuous variables with a normal distribution were reported as means ± standard deviation and analyzed using the independent samples *t*-test or analysis of variance. For continuous variables that did not follow a normal distribution, results were expressed as medians with interquartile ranges and compared using the Mann–Whitney U test or the Kruskal–Wallis H test. The relationship between independent variables (renal vascular variations or normal renal vasculature) and dependent variables (resistant vs. controlled hypertension) was evaluated using logistic regression analysis, with the strength of association represented by the odds ratio (OR). A *p*-value of less than 0.05 was considered statistically significant.

## 3. Results

### 3.1. Renal Vascular Characteristics

The contrast-enhanced CT imaging of the renal vasculature revealed anatomical variations in 64 (25%) of the 256 participants. These variations were more common in patients with resistant HTN, observed in 49 (38.3%) of 128 patients, compared to 15 (11.7%) of 128 patients with controlled HTN (*p* < 0.001).

A variety of abnormalities in the renal vasculature were identified, with unilateral accessory renal arteries being the most frequent finding in both groups. However, this abnormality had a significantly higher prevalence in the resistant HTN group. Other more common findings included bilateral accessory renal arteries, the presence of two renal veins in a single kidney, and Nutcracker syndrome. The classic form of this syndrome refers to the compression of the left renal vein between the superior mesenteric artery and the abdominal aorta [18]. In our study, four patients presented with this form of Nutcracker syndrome, while one patient—a 28-year-old male—was diagnosed with the rarer right-sided Nutcracker syndrome.

Other types of kidney vessel anomalies were also more prevalent in patients with resistant HTN. However, a statistical analysis did not reveal significant differences between patients with resistant and controlled HTN regarding the prevalence of specific types of abnormalities. Among the 64 patients with congenital renal vascular abnormalities, 17 (26.6%) had two or more malformations. Table 1 summarizes the types and prevalence of renal vascular variations observed in our study.

**Figure 1:**
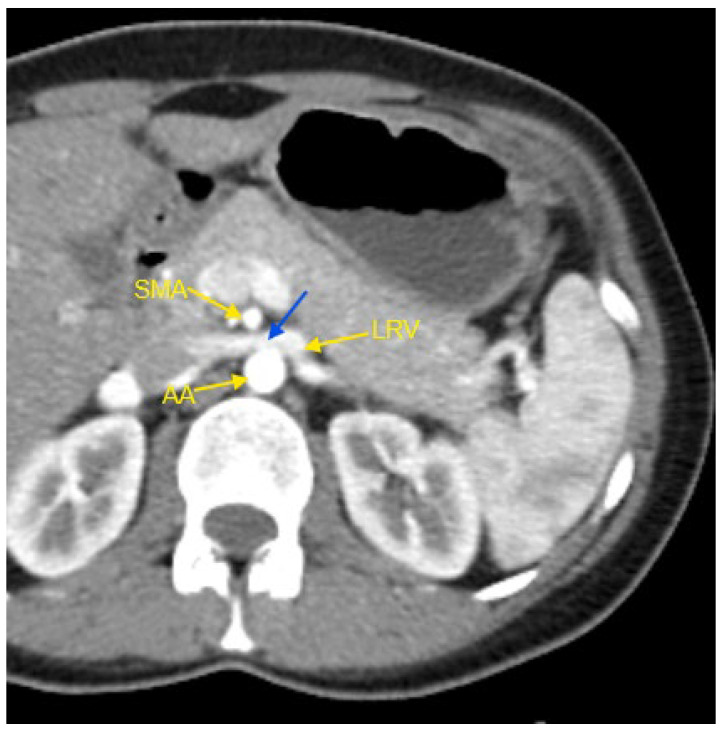
Contrast-enhanced CT imaging of a 44-year-old female patient with high blood pressure refractory to treatment, revealed characteristic findings consistent with classic Nutcracker syndrome. In the presented image, the abdominal aorta, the superior mesenteric artery, and the left renal vein are indicated by yellow arrows. The compressed segment of the left renal vein is highlighted by a blue arrow. AA—abdominal aorta; CT—computed tomography; LRV—left renal vein; SMA—superior mesenteric artery.

**Figure 2:**
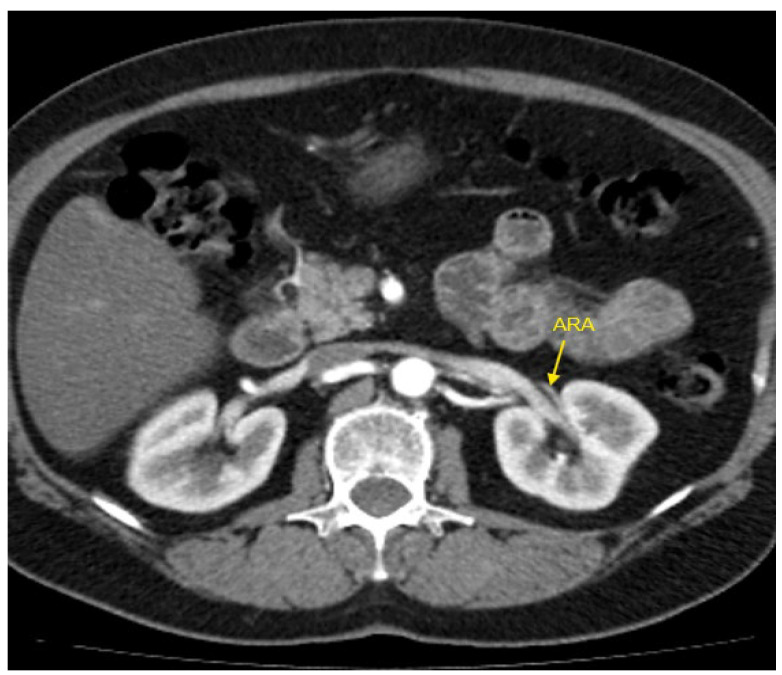
Contrast-enhanced CT imaging of a 47-year-old female patient with refractory hypertension revealed an accessory renal artery in the left kidney, shown by the yellow arrow. ARA—accessory renal artery; CT—computed tomography.

**Figure 3:**
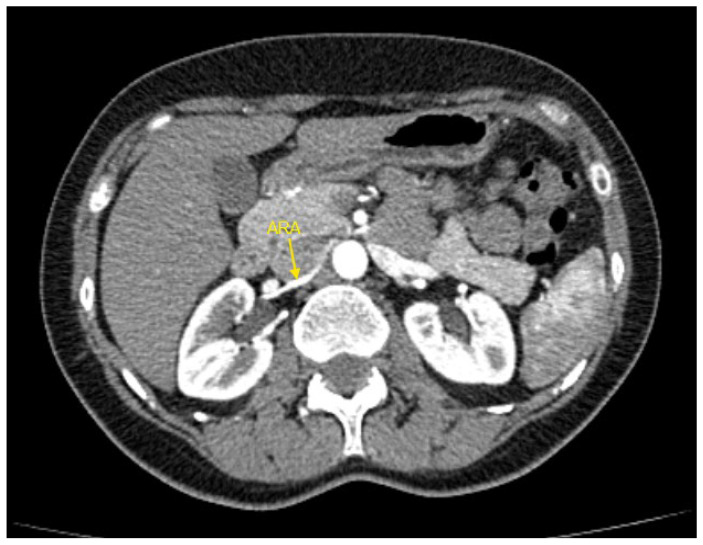
Contrast-enhanced CT imaging of a 49-year-old female hypertensive patient failing to achieve blood pressure control by 4 drugs from different classes demonstrated an accessory renal artery in the right kidney, indicated by the yellow arrow. ARA—accessory renal artery; CT—computed tomography.

**Figure 4:**
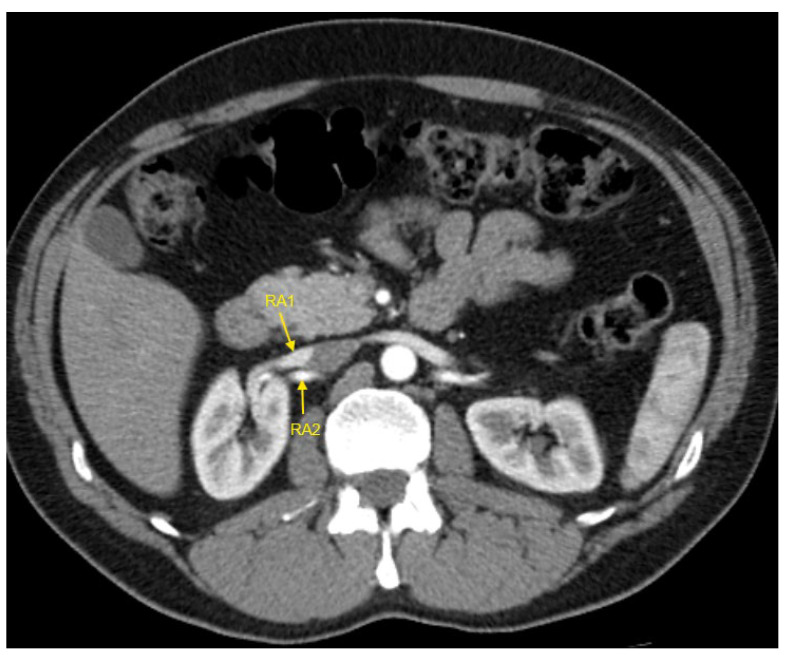
Contrast-enhanced CT imaging of a 43-year-old male patient with true resistant hypertension showed the presence of two renal arteries in the right kidney, indicated by the yellow arrows. CT—computed tomography; RA1—first renal artery; RA2—second renal artery.

**Figure 5:**
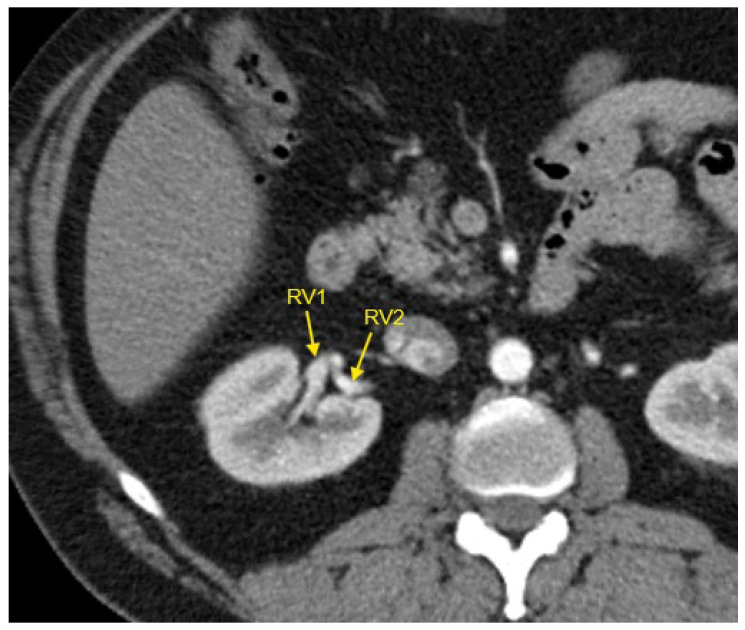
Contrast-enhanced CT imaging of a 44-year-old male hypertensive patient with difficult-to-control HTN revealed two renal veins in the right kidney, marked by the yellow arrows. CT—computed tomography; HTN—arterial hypertension; RV1—first renal vein; RV2—second renal vein.

Figure 1, Figure 2, Figure 3, Figure 4 and Figure 5 show examples of some renal vascular abnormalities we identified in our study population.

### 3.2. Demographic and Clinical Characteristics

Table 2 compares the clinical features of hypertensive patients with congenital renovascular disorders and those with normal kidney vasculature. Men and women were equally represented in the overall study population and in the two comparison groups (vascular anomalies vs. normal vessels). Hypertensive patients with renal vascular variations were younger, with a median age difference of 9 years compared to those with normal renal vasculature.

Therapy-resistant HTN was significantly more prevalent among patients with anatomical renal vascular variations than among those with normal kidney vasculature. Among participants with renal vascular abnormalities, 76.6% had resistant HTN, while only 23.4% had controlled HTN. Severe hypertension was also more common in the group with renal vascular variations.

Regarding concomitant risk factors and comorbidities, most were equally represented in both groups, with the notable exception of active smoking, which was three times more common among patients with congenital renal vascular abnormalities.

### 3.3. Laboratory Investigations

Table 3 displays the basic laboratory parameters of the study participants. Patients with renal vascular variations had higher potassium levels, compared to patients with controlled HTN. For all other laboratory parameters, both groups were comparable.

### 3.4. BP Measurement

Table 4 presents the office blood pressure and heart rate assessment, and 24 h ABPM readings, for the study population. Patients with congenital renal vascular anatomic variations had significantly higher office BP and ABPM values compared to those with normal kidney vessels.

**Figure 6:**
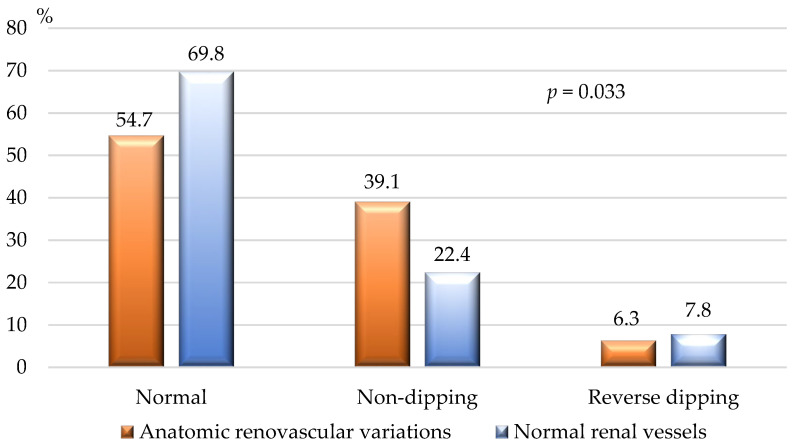
Dipping state of patients with renal vascular variations and normal renal vasculature. Normal dipping—physiological 10–20% lowering of SBP and DBP in the nighttime compared to the daytime values; non-dipping—fall of the nighttime SBP and/or DBP by only 1–9% to the daytime values; reverse dipping—elevation of the nighttime SBP and/or DBP to the daytime values; DBP—diastolic blood pressure; SBP—systolic blood pressure; *p*—*p*-value for statistical significance.

Figure 6 demonstrates the BP dipping status, assessed by 24 h ABPM. Non-dipping state was significantly more common among individuals with congenital renal vascular variants.

### 3.5. Impact of Congenital Renal Vascular Variations on HTN Control

Table 5 summarizes the results of the logistic regression analysis conducted to assess the association between congenital renal vascular variations and the development of resistant HTN in our study. A significant positive association was identified, with renal vascular variations increasing the overall likelihood of refractory-to-treatment HTN by approximately 4.7-fold.

## 4. Discussion

This study aimed to assess the association between renal vascular variations and the development of resistant HTN. Congenital abnormalities in renal vasculature are among the most common vascular malformations, yet their clinical significance remains poorly understood [2,4,5,7,9]. In our study, renal vascular variations were identified in 25% of participants, with no gender differences in prevalence, and this finding aligns with previous reports [1,2,5,6,9,19,20].

The CT imaging performed in our study demonstrated 19 distinct types of renal vascular abnormalities, with arterial and venous variations being almost equally represented. We did not observe a predominant localization in the right or left kidney. In the study of García-Barrios et al., arterial vascular malformations were observed more frequently in the right kidney, and venous vessel abnormalities affected equally both kidneys [1]. Similar findings have been described by Ugurel et al. and Özkan et al. in two renal CT angiographic studies involving 100 and 855 patients, respectively [21,22].

The most common renal vascular abnormalities in our study were unilateral or bilateral accessory renal arteries, the presence of two renal veins in one kidney, and Nutcracker syndrome. At least one accessory artery, with or without additional abnormalities, was present in 19.3% of our patients. Similarly, García-Barrios et al. reported accessory polar renal arteries as the most frequent renovascular variation, observed in 56.3% of cases, although their study involved only 16 dissected kidneys [1]. Satyapal et al. and Khamanarong et al. found accessory polar renal arteries in 27.7% and 17% of cases, respectively [3,7]. A direct comparison between our findings and these studies is challenging due to the differences in study populations, as we exclusively enrolled hypertensive patients, whereas the other cohorts were more heterogeneous in clinical characteristics and inclusion/exclusion criteria.

We hypothesize that renal vascular variations may contribute to the development of HTN, a notion supported by other authors, such as Tremblay and Sanghvi [12,13]. However, to date, no dedicated clinical investigations have provided direct evidence to confirm this hypothesis. Establishing a causal relationship is inherently challenging due to several factors: 1. Hypertensive disease is the most common cardiovascular condition globally, affecting approximately 1.5 billion individuals [14,15]; 2. Primary HTN has a heterogeneous pathogenesis, influenced by a multitude of factors including age, genetic predisposition (>1000 genes are implicated in BP regulation), obesity, increased salt intake and others [14,15]; 3. Primary HTN increasingly affects younger populations, and most patients have multiple risk factors for high BP [14,15]. The Seventh Report of the Joint National Committee on Prevention, Detection, Evaluation, and Treatment of High Blood Pressure showed that 55-year-old adults with normal blood pressure have a ~90% lifetime risk of developing arterial hypertension [23]. This statistic underscores the inevitability of HTN in the general population, irrespective of renal vascular variations. Consequently, our study focused on investigating the association between congenital renal vascular abnormalities and resistant HTN—a clinically and practically significant subset of HTN.

In our research, renovascular variations were significantly more common in patients with treatment-resistant hypertension compared to those who achieved target BP values through standard treatment regimens. A logistic regression analysis demonstrated that congenital renal vascular abnormalities overall increased the odds of developing resistant HTN by more than 4.5-fold, with the most common abnormalities in our study—accessory renal arteries or veins—showing an increase in odds by more than 6-fold. We found no other studies with a similar design and cohort to allow a direct comparison. Kasprzycki et al. observed frequent accessory renal arteries in patients with resistant HTN but provided no specific data for comparison [11]. Current guidelines for the diagnosis and management of HTN acknowledge that certain renal vascular diseases can cause secondary HTN, often resistant to treatment [14,15]. However, none of these documents mentions renal vascular variations as potential etiologies for secondary or resistant HTN [14,15].

In our cohort, patients with anatomical renal vascular variants and resistant HTN were middle-aged (median age ~54 years) and had no concomitant conditions or diseases that could explain their resistance to standard pharmacological treatment. Good adherence to therapy was an inclusion criterion, and secondary HTN due to other causes was an exclusion criterion. Thus, we propose that renal vascular variations, or at least some of them, may contribute to the development of resistant HTN.

The pathophysiology of resistant hypertension is complex. It involves increased sympathetic activity, elevated levels of angiotensin II, aldosterone, vasopressin, endothelin-1 and other vasoconstrictors, and/or the decreased production of vasodilator substances such as nitric oxide, prostaglandins, bradykinin, and others [14,15,24,25,26]. The imbalanced effects of these factors lead to increased peripheral vascular resistance and arterial stiffness, sodium and fluid overload, and progressive hypertensive-mediated organ damage, particularly cardiorenal injury [14,24,25,26]. The precise mechanisms underlying BP elevation in patients with renal vascular variations remain unclear [10,12,14,15]. We hypothesize that renovascular abnormalities contribute to resistant hypertension primarily through the activation of the renin–angiotensin–aldosterone system. Increased sympathetic activity is unlikely to play a significant role, as patients with high sympathetic activity typically exhibit resting heart rates ≥80 beats/min, while the median resting heart rate in our cohort was 74 beats/min. Although the increased secretion of other vasoconstrictors (e.g., endothelins, vasopressin, others) cannot be excluded, we consider RAAS activation the most probable pathophysiological mechanism.

Renovascular abnormalities may also contribute to systemic endothelial activation, leading to vasculopathy and hypertension. This is supported by studies on vascular and endothelial dysfunction markers, such as CD93 and VCAM-1, which have been associated with both nephropathy and cardiopathy—further reinforcing the broader implications of vascular anomalies in hypertension development [27,28].

Our study is a pilot investigation that seeks to address unresolved questions about the clinical significance of anatomical renal vascular variations, particularly their prevalence and association with resistant HTN. Large-scale future studies are necessary to evaluate the impact of different variations on BP control and to elucidate the precise mechanisms contributing to resistant HTN. These insights could improve treatment strategies and BP control in affected patients.

### Study Limitations

The weaknesses of our study are as follows: 1. The small sample size and single-center design restrict the generalizability of our findings. 2. We assessed the association between renal vascular abnormalities and resistant HTN collectively, without analyzing the impact of specific types beyond accessory arteries and veins. 3. Pathogenic mechanisms underlying resistant HTN in the presence of renal vascular variations were not investigated through laboratory tests or other instrumental methods. 4. Our cohort consisted exclusively of hospitalized hypertensive patients, who may differ from ambulatory populations. 5. Our study was cross-sectional, not prospective; therefore, we do not have follow-up data on the patients after treatment was reconsidered during hospitalization, as they were monitored in outpatient medical centers. 6. The data on kidney size were only descriptive and not suitable for a precise statistical analysis.

## 5. Conclusions

Anatomical renal vascular variations were highly prevalent in our hypertensive cohort. The most common abnormalities were unilateral or bilateral accessory renal arteries, double renal veins, and Nutcracker syndrome. These variations were strongly associated with the development of treatment-resistant HTN. Future large-scale studies are needed to evaluate the impact of specific vascular variations on blood pressure control and elucidate their role in resistant HTN pathophysiology, which may lead to improved management strategies for these patients.

## Figures and Tables

**Table 1:** Comparison of the incidence and type of anatomical variations in patients with controlled vs. resistant HTN.

Anatomical Renal Vascular Variations	Total *n* = 256	Controlled HTN *n* = 128	Resistant HTN *n* = 128	*p*
Normal anatomy, *n* (%)	192 (75.0%)	113 (88.3%)	79 (61.7%)	0.005
Accessory right-sided renal artery, *n* (%)	16 (6.3%)	4 (3.1%)	12 (9.4%)	0.04
Accessory left-sided renal artery, *n* (%)	18 (7.0%)	4 (3.1%)	14 (11%)	0.04
Accessory renal artery of both kidneys, *n* (%)	4 (1.6%)	1 (0.8%)	3 (2.4%)	0.321
Accessory renal artery of the right kidney + 2 renal veins of the right kidney, *n* (%)	4 (1.6%)	2 (1.6%)	2 (1.6%)	1.000
Accessory renal artery of both kidneys + 2 renal veins of the right kidney, *n* (%)	1 (0.4%)	0 (0%)	1 (0.8%)	0.318
Accessory renal vein of the right kidney, *n* (%)	1 (0.4%)	0 (0%)	1 (0.8%)	0.318
Nutcracker syndrome—left-sided, *n* (%)	4 (1.6%)	1 (0.8%)	3 (2.3%)	0.321
Nutcracker syndrome—right-sided, *n* (%)	1 (0.4%)	0 (0%)	1 (0.8%)	0.318
Accessory and aberrant right renal arteries + accessory left renal artery + left-sided Nutcracker syndrome, *n* (%)	1 (0.4%)	0 (0%)	1 (0.8%)	0.318
Left-sided double kidney with separate renal arteries, veins and ureters, *n* (%)	1 (0.4%)	0 (0%)	1 (0.8%)	0.318
Aberrant lower-pole artery of the right kidney + right main renal artery stenosis, *n* (%)	2 (0.8%)	1 (0.8%)	1 (0.8%)	1.000
Accessory right-sided renal artery + left renal vein stenosis, *n* (%)	1 (0.4%)	0 (0%)	1 (0.8%)	0.318
Accessory left renal artery + right main renal artery stenosis + 2 renal veins and double draining system of the right kidney, *n* (%)	1 (0.4%)	0 (0%)	1 (0.8%)	0.318
Accessory renal vein of the right kidney + calcification of the ostia of both renal arteries, *n* (%)	1 (0.4%)	0 (0%)	1 (0.8%)	0.318
Double right kidney + accessory lower-pole right renal artery, *n* (%)	1 (0.4%)	0 (0%)	1 (0.8%)	0.318
Early bifurcation of the right renal artery, *n* (%)	2 (0.8%)	1 (0.8%)	1 (0.8%)	1.000
Multiple aneurisms of the right renal artery, *n* (%)	1 (0.4%)	0 (0%)	1 (0.8%)	0.318
Retroaortic left renal vein, *n* (%)	2 (0.8%)	0 (0%)	2 (1.6%)	0.159
Trifurcation of right renal artery with early separation of the branches, *n* (%)	2 (0.8%)	1 (0.8%)	1 (0.8%)	1.000

The chi-square test was used to compare the incidence of different types of renal vascular variations in patients with controlled versus resistant hypertension; *p*—*p*-value for statistical significance.

**Table 2:** Patient demographic and clinical characteristics at inclusion in the study.

	Total *n* = 256	Renal Vascular Variations *n* = 64	Normal Renal Vasculature *n* = 192	*p*
Age (years), median (IQR)	61.0 (51.0–69.0)	54.0 (46.0–65.8)	63.0 (54.0–71.0)	<0.001
Gender, *n* (%)				
Males	130 (50.8%)	32 (50%)	98 (51.0%)	0.886 ^#^
Females	126 (49.2%)	32 (50%)	94 (49.0%)	
Resistant HTN, *n* (%)	64 (25.0%)	49 (76.6%)	79 (41.1%)	<0.001
HTN grade, *n* (%)				
Mild	74 (28.9%)	3 (4.7%)	71 (37.0%)	<0.001 ^#^
Moderate	88 (34.4%)	23 (35.9%)	65 (33.9%)	
Severe	94 (36.7%)	38 (59.4%)	56 (29.2%)	
HTN stage, *n* (%)				
1st stage	79 (30.9%)	22 (34.4%)	57 (29.7%)	0.780 ^#^
2nd stage	109 (40.6%)	26 (40.6%)	83 (43.2%)	
3rd stage	68 (26.6%)	16 (25.0%)	52 (27.1%)	
CKD, *n* (%)	126 (49.2%)	32 (50.0%)	94 (49.0%)	0.585
Overweight/obesity, *n* (%)	105 (41.0%)	28 (43.8%)	77 (40.1%)	0.320
Dyslipidemia, *n* (%)	81 (31.6%)	18 (28.1%)	63 (32.8%)	0.296
Type 2 diabetes, *n* (%)	38 (14.8%)	10 (15.6%)	28 (14.6%)	0.272
Ischemic heart disease, *n* (%)	31 (12.1%)	7 (10.9%)	24 (12.5%)	0.467
Post-stroke, *n* (%)	11 (4.3%)	2 (3.1%)	9 (4.7%)	0.516
PAD, *n* (%)	11 (5.5%)	3 (6.4%)	8 (5.3%)	0.723
Heart failure, *n* (%)	38 (14.8%)	7 (10.9%)	31 (16.1%)	0.326
Smoking, *n* (%)				
Active	53 (20.7%)	28 (43.8%)	25 (13.0%)	<0.001 ^#^
Ex-smoker	16 (6.3%)	4 (6.3%)	12 (6.3%)	
Alcohol consumption *	33 (12.9%)	12 (18.8%)	21 (10.9%)	0.131

CKD—chronic kidney disease; HTN—arterial hypertension; IQR—interquartile range; HTN grading: Mild—SBP 140–159 and/or DBP 90–99 mmHg; Moderate—SBP 160–179 and/or DBP 100–109 mmHg; Severe—SBP ≥180 and/or DBP ≥180 mmHg; Obesity—body mass index 25–29.9 kg/m^2^; Overweight—body mass index ≥30 kg/m^2^; PAD—peripheral arterial disease; * ≥14 units per week for males and ≥8 units per unit for females (1 unit = 25 mL of standard alcohol drink with 40.0% alcohol content or 125 mL of wine with 12.0% alcohol content or 250 mL beer with 5.0% alcohol content); ^#^—the comparison is between the groups (renal vascular variations versus controlled normal renal vasculature); *p*—*p*-value for statistical significance.

**Table 3:** Basic laboratory parameters of the study population.

Parameter	Total *n* = 256	Renal Vascular Variations *n* = 64	Normal Renal Vasculature *n* = 192	*p*
Potassium, mmol/L, median (IQR)	4.6 (4.2–4.9)	4.7 (4.3–4.9)	4.6 (4.5–4.9)	0.007
Sodium, mmol/L, median (IQR)	142 (139–145)	141 (138–145)	142 (139–145)	0.286
Hemoglobin, g/L, median (IQR)	148 (136–158)	147 (134–160)	148 (137–157)	0.944
Hematocrit, L/L, median (IQR)	0.44 (0.42–0.46)	0.45 (0.41–0.47)	0.44 (0.42–0.46)	0.353
Fasting glucose, mmol/L, median (IQR)	5.3 (4.8–6.2)	5.2 (4.8–6.1)	5.4 (4.9–6.2)	0.361
Creatinine, µmol/L, median (IQR)	76 (66–92)	77 (68–91)	76 (66–92)	0.960
eGFR, mL/min/1.73 m^2^, median (IQR)	92 (75–101)	95 (76–105)	92 (73–100)	0.129

eGFR—estimated glomerular filtration rate (according to the equation recommended by the 2021 Guidelines of CKD Epidemiology Collaboration Group) [16]; IQR—interquartile range; *p*—*p*-value for statistical significance.

**Table 4:** Office BP measurement with pulse pressure calculation, 24 h ABPM, and office-measured HR.

Characteristics	Total *n* = 256	Renovascular Variations *n* = 64	Normal Renal Vasculature *n* = 192	*p*
Office SBP (mm Hg), median (IQR)	139.0 (130.0–150.0)	147.0 (138.5–160.0)	135.5 (130.0–150.0)	<0.001
Office DBP (in mm Hg), median (IQR)	85.0 (80.0–90.0)	90.0 (83.5.0–95.0)	85.0 (80.0–90.0)	<0.001
Pulse pressure (in mm Hg), median (IQR)	51.0 (50.0–60.0)	52.0 (50.0–65.0)	51.0 (48.0–60.0)	0.062
Daytime ABPM SBP (in mm Hg), median (IQR)	134.0 (128.5–145.0)	142.0 (135.0–149.0)	133.0 (126.5–142.0)	<0.001
Daytime ABPM DBP (in mm Hg), median (IQR)	84.0 (78.0–90.0)	89.0 (82.5–94.0)	83.0 (76.0–90.0)	<0.001
Nighttime ABPM SBP (in mm Hg), median (IQR)	123.0 (115.0–137.0)	131.0 (124.0–142.0)	119.0 (115.0–134.0)	<0.001
Nighttime ABPM DBP (in mm Hg), median (IQR)	74.0 (65.0–85.5)	82.0 (73.5–90.0)	69.0 (65.0–82.5)	<0.001
24 h ABPM SBP (in mm Hg), median (IQR)	132.0 (122.0–141.0)	139.0 (132.0–145.5)	127.0 (121.0–139.0)	<0.001
24 h ABPM DBP (in mm Hg), median (IQR)	79.0 (71.0–88.0)	85.0 (78.0–92.0)	77.0 (70.0–87.0)	<0.001
Heart rate (beats/min.), median (IQR)	74 (67–80)	75 (70–85.0)	74 (67.0–80.0)	0.402

ABPM—ambulatory blood pressure monitoring; DBP—diastolic blood pressure; HR—heart rate; IQR—interquartile range; SBP—systolic blood pressure; *p*—*p*-value for statistical significance.

**Table 5:** Association of renal vascular variations with resistant HTN.

Variable	OR	95% CI for OR		
		Lower Limit	Upper Limit	*p*
Overall renal vascular variation(s)	4.673	2.449	8.913	<0.001
Accessory renal artery(ies)	6.373	2.744	14.799	<0.001
Accessory renal vein(s)	6.153	1.240	12.541	0.026
Normal renal vessels	0.214	0.112	0.408	<0.001

CI—confidence interval; OR—odds ratio; *p*—*p*-value for statistical significance.

## Data Availability

Data supporting reported results are available and can be provided by Stefan Naydenov; email: snaydenov@gmail.com.
